# Important role of CCR2 in a murine model of coronary vasculitis

**DOI:** 10.1186/1471-2172-13-56

**Published:** 2012-10-17

**Authors:** Hernan G Martinez, Marlon P Quinones, Fabio Jimenez, Carlos Estrada, Kassandra M Clark, Kazuo Suzuki, Noriko Miura, Naohito Ohno, Sunil K Ahuja, Seema S Ahuja

**Affiliations:** 1 Department of Medicine (MC 7870), University of Texas Health Science Center at San Antonio, 7703 Floyd Curl Drive, San Antonio, TX, 78229-3900, USA; 2 South Texas Veterans Health Care System, Audie L. Murphy Division, San Antonio, TX, 78229-3900, USA; 3 Department Psychiatry, University of Texas Health Science Center at San Antonio (UTHSCSA), San Antonio, TX, 78229-3900, USA; 4 Inflammation Program, Department of Immunology, Chiba University Graduate School of Medicine, Chiba, 260-8670, Japan; 5 Laboratory for Immunopharmacology of Microbial Products, School of Pharmacy, Tokyo University of Pharmacy and Life Science, Hachioji Tokyo, 192-0392, Japan; 6 The Veterans Administration Center for Aids and HIV-1 infection, South Texas Veterans Health Care System, San Antonio, TX, USA

**Keywords:** CCR2, Coronary vasculitis, Treg, Treg/Th17 imbalance

## Abstract

**Background:**

Chemokines and their receptors play a role in the innate immune response as well as in the disruption of the balance between pro-inflammatory Th17 cells and regulatory T cells (Treg), underlying the pathogenesis of coronary vasculitis in Kawasaki disease (KD).

**Results:**

Here we show that genetic inactivation of chemokine receptor (CCR)-2 is protective against the induction of aortic and coronary vasculitis following injection of *Candida albicans* water-soluble cell wall extracts (CAWS). Mechanistically, both T and B cells were required for the induction of vasculitis, a role that was directly modulated by CCR2. CAWS administration promoted mobilization of CCR2-dependent inflammatory monocytes (iMo) from the bone marrow (BM) to the periphery as well as production of IL-6. IL-6 was likely to contribute to the depletion of Treg and expansion of Th17 cells in CAWS-injected *Ccr2*^*+/+*^ mice, processes that were ameliorated following the genetic inactivation of CCR2.

**Conclusion:**

Collectively, our findings provide novel insights into the role of CCR2 in the pathogenesis of vasculitis as seen in KD and highlight novel therapeutic targets, specifically for individuals resistant to first-line treatments.

## Background

Kawasaki disease (KD) is a form of vasculitis that predominantly affects infants and toddlers, and specifically targets coronary arteries, resulting in increased risk of myocardial ischemia, heart disease and sudden death [1]. Although most studies describe KD in Japanese children, KD occurs in children of all ethnicities and geographic regions [2]. In the United States, KD remains the leading cause of acquired heart disease, affecting up to 4,000 children each year [3]. Research in KD etiology and pathogenesis addresses major knowledge gaps. There is a real need to identify novel therapeutic targets for KD since 25% of patients are resistant to intravenous immunoglobulin infusion (IVIG), the most common and effective treatment for KD. Moreover, the administration of IVIG is quite expensive and used only for symptomatic patients [4].

Increasing clinical and experimental evidence suggests that abnormal immune responses to infectious agent(s) are a key component of disease initiation [5,6]. The imbalanced immune response fueling KD is thought to encompass both the innate and adaptive immunity, as suggested by the elevation of pro-inflammatory mediators and increased activation of lymphocytes in KD patients [6].

In this study, we used a coronary vasculitis model, based on the injection of a water-soluble fraction of *Candida albicans* (CAWS) in C57BL/6J mice (WT) [7,8]. In this model system, we investigated the inflammatory mediators, including chemokines and chemokine receptors, responsible for orchestrating leukocyte migration and other immune processes in the pathogenesis of a form of coronary vasculitis that resembles KD [5,9]. Four lines of evidence suggested that the CC chemokine ligand 2 (CCL2) – CCR2 axis would play a role in coronary vasculitis. First, CCR2 is required for monocyte/macrophage migration and activation [10], a population of cells thought to promote tissue damage in KD [6]. Second, previous reports indicated that there is marked up-regulation of chemokine CCL2 [11] levels during the acute phase of KD for which the receptor is CCR2 [11]. Third, evidence to the occurrence of KD is linked to common genetic variants in the chemokine receptor gene-cluster CCR3–CCR2–CCR5 [5]. Finally, diverse experimental models implicate CCR2 in the establishment of tolerance or development of autoimmunity [12]. Moreover increasing evidence points towards the loss of regulatory mechanisms (depletion of regulatory T cells, Treg), along with amplification of T cell driven inflammation (expansion of T helper cells producing IL-17, Th17), in KD [13,14]. Our research highlights the critical role of CCR2 in the pathogenesis of coronary vasculitis seen in KD and identifies this chemokine receptor as an important determinant of the Treg/Th17 balance which may be critical for disease initiation and maintenance [13,15].

## Results

### *Ccr2*^*−/−*^ mice are protected against CAWS-induced vasculitis

**Figure 1:**
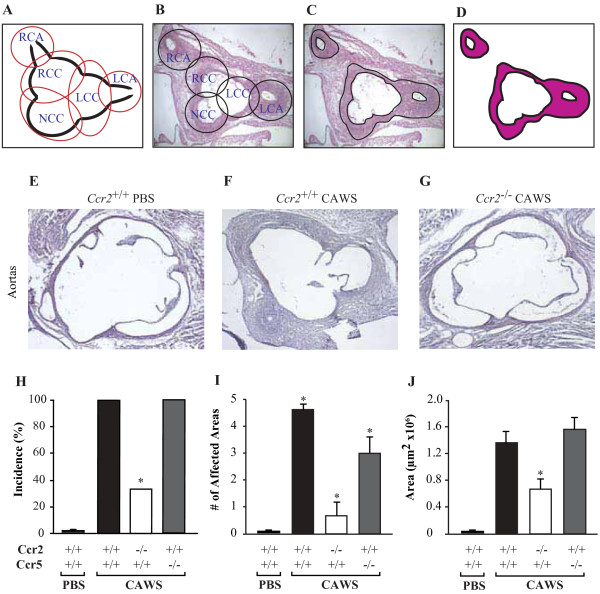
***Ccr2***^***−/−***^**mice, but not *Ccr5***^***−/− ***^**mice, are resistant to coronary/aortic inflammation after CAWS administration.** (**A**-**B**) Representative diagram and histopathology of the five areas quantified for inflammation at the aortic root that included: right coronary artery (RCA), left coronary artery (LCA), right coronary cuspid (RCC), left coronary cuspid (LCC), and non-coronary cuspid (NCC). (**C**-**D**) Quantification of the inflamed area at the level of the root of the aorta. (**E**) Normal vascular structures showing the coronaries at the level of the root of the aorta in PBS-treated *Ccr2*^*+/+*^ (WT) mice. (**F**) Coronary and aortic inflammation in *Ccr2*^*+/+*^ mice after a full cycle of CAWS. (**G**) Decreased inflammation in *Ccr2*^*−/−*^ mice, at the level of the root of the aorta, after CAWS administration (all pictures have a 4X magnification and were cropped for comparison purposes). (**H**) Incidence of coronary/aortic inflammation between *Ccr2*^*+/+*^, *Ccr2*^*−/−*^, and *Ccr5*^*−/−*^ mice, after CAWS administration. (**I**) Number of areas affected in the coronary and aortic wall. (**J**) Total area inflamed (μm^2^) in each group of mice receiving two cycles of CAWS. Each bar represents the mean ± SE from a representative experiment out of 3 performed with 6–8 mice per group.

We observed that injection of CAWS following the protocol described (Additional file 1) induces vasculitis in the coronary arteries and aortic root (Figure 1A-G) with histological changes that have been classified as granulomatous proliferative inflammation [8]. With this type of inflammation the normal structure of the arteries is destroyed (the internal elastic lamina, external elastic lamina, and the smooth muscle layer of the tunica media are severely damaged). Indeed in agreement with previous findings, there was a prominent infiltration of mononuclear cells, such as histiocytes, fibroblasts, etc., and neutrophils (Figure 1 and data not shown).

As previously described [7,8] after CAWS injection we quantified vasculitis severity, by enumerating five anatomical sites at the level of the aortic root (Figure 1A and B), as well as measuring the inflamed aortic wall area (Figure 1C and D). Understanding that incidence was defined as having one or more inflamed areas, 100% of *Ccr2*^*+/+*^ mice developed coronary/aortic inflammation following CAWS injection compared to PBS controls and *Ccr2* null mice (Figure 1E-H), had a mean of 4–5 areas inflamed in contrast to a mean of 0.8 areas in *Ccr2*^*−/−*^ mice (Figure 1I), and the area of inflammation was several folds higher (Figure 1J).

Highlighting the specificity of the protective phenotype afforded by CCR2 inactivation, 100% of *Ccr5*^*−/−*^ mice exposed to CAWS developed coronary vasculitis with the same area of inflammation seen in wild type mice (Figure 1H and J); and exhibiting only a small reduction in the number of affected areas (mean = 3, Figure 1I).

### Decrease inflammatory infiltrate in the heart of *Ccr2*^*−/−*^ mice injected with CAWS

**Figure 2:**
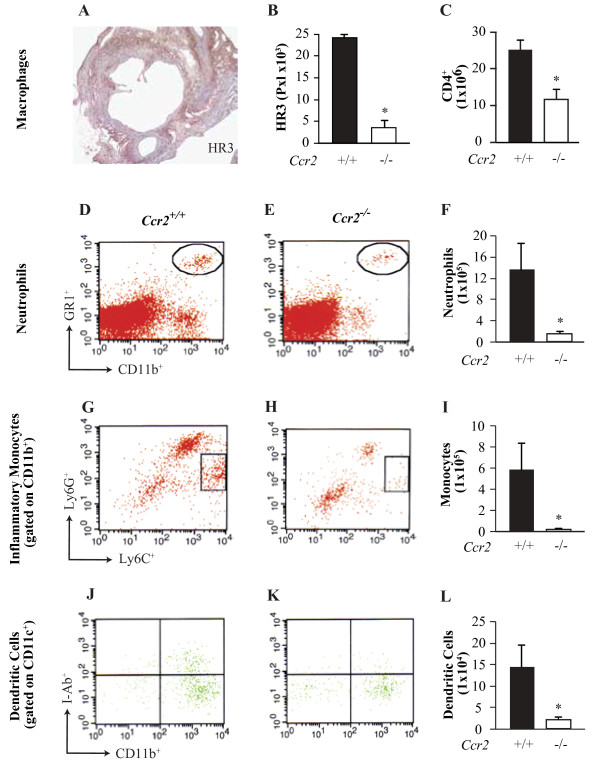
**Decreased inflammatory infiltrate in the hearts of *Ccr2***^***−/− ***^**mice injected with CAWS.** (**A**) Immunohistochemical staining of macrophages using the marker ER-HR3 in *Ccr2*^*+/+*^ mice and (**B**) quantification (pixels) of infiltration, at the level of the root of the aorta. (**C**) Absolute number of T cells (CD4^+^). (**D**) Representative FACS plots of neutrophils as defined by the expression of CD11b^+^ and Gr-1^+^ in *Ccr2*^*+/+*^ and (**E**) *Ccr2*^*−/−*^ mice, with (**F**) absolute number of neutrophils. (**G**) FACS of iMo (CD11b^+^, Ly6C^hi^, Ly6G^int^) in *Ccr2*^*+/+*^ mice and (**H**) *Ccr2*^*−/−*^ mice, with (**I**) absolute number of iMo. (**J**) Representative plots of dendritic cells (CD11b^+^, CD11c^+^, I-Ab^+^) in *Ccr2*^*+/+*^ mice and (**K**) *Ccr2*^*−/−*^ mice. (**L**) Absolute number of dendritic cells. A-L represents data collected from the heart at the level of the aortic root after two cycles of CAWS. Each bar represents the mean ± SE from a representative experiment with 6–8 mice per group.

Immunohistochemistry at the level of the aortic root revealed that CAWS-injected *Ccr2*^*−/−*^ mice had less macrophages present in the vessel wall compared with CAWS-injected *Ccr2*^*+/+*^ mice, (Figure 2A-B and Additional file 2). Also, compared with CAWS-injected *Ccr2*^*+/+*^ mice, FACS analysis of cell suspensions arising from the affected area (root of the aorta) revealed that CAWS-injected *Ccr2*^*−/−*^ mice had significantly lower proportions of CD4^+^ T cells (Figure 2C), neutrophils (Figure 2D-F), inflammatory monocytes (iMo; Figure 2G-I), and activated dendritic cells (Figure 2J-L), (p <0.05). Paralleling the results described above, myeloperoxidase (MPO) levels in CAWS-injected *Ccr2*^*+/+*^ mice were significantly higher in serum from CAWS-injected mice, compared to PBS-injected mice (Additional file 3). As expected, due to the milder vasculitis phenotype in *Ccr2*^*−/−*^ mice, serum MPO level post-injection in these mice was lower than in *Ccr2*^*+/+*^ mice (Additional file 3).

### *Ccr2*^*−/−*^ T and B cells are partially sufficient for protection against CAWS-induced coronary vasculitis

**Figure 3:**
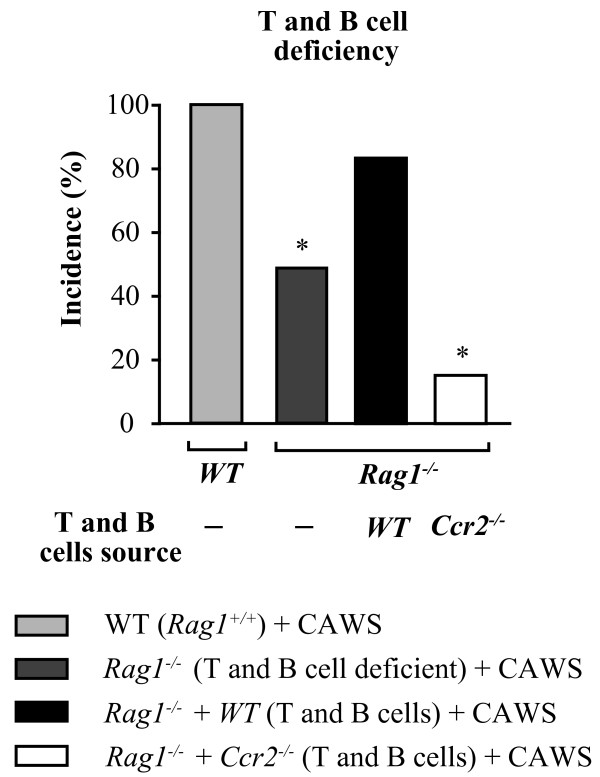
**Contribution of T and B cells to the development of coronary/aortic inflammation after CAWS administration.** Incidence of coronary inflammation after CAWS administration in *Rag1*^*−/−*^ mice receiving T and B cells from *Ccr2*^*+/+*^ mice or *Ccr2*^*−/−*^ mice. Each bar represents the mean ± SE from a representative experiment with 6–8 mice per group.

Supporting the contribution of adaptive immunity in CAWS-induced vasculitis, we found that mice lacking mature T and B lymphocytes (*Rag1*^*−/−*^) had a lower incidence (50%) and decreased number of affected areas compared with WT mice (Figure 3 and data not shown). However, *Rag1*^*−/−*^ mice reconstituted with WT T and B cells had a similar phenotype as the WT mice (incidence 85%, Figure 3). But most importantly, *Rag1*^*−/−*^ mice reconstituted with T and B cells from *Ccr2*^*−/−*^ mice had significantly lower incidence of CAWS-induced vasculitis (16%) compared with WT mice (Figure 3).

Looking at the phenotype of mice only lacking mature T cells (nude mice) we found that compared with WT controls, nude mice had the same disease incidence and severity after CAWS administration (data not shown).

CAWS administration in WT mice was linked to the elicitation of antibodies against MPO, anti-CAWS IgG1, and IgG2a (Additional file 4 A-C). Interestingly, *Ccr2*^*−/−*^ mice that received CAWS administration had lower levels of potentially pathogenic anti-MPO antibodies, compared with WT mice (Additional file 4 A). Nevertheless, bringing into question the pathogenic role of anti-MPO and anti-CAWS antibodies, we found that similar to the WT mice (n= 10), 100% of B cell-deficient mice (*Igh*^*−/−*^ mice; n= 8) developed vasculitis, after CAWS administration (data not shown). Together, the data in Figure 3 using *Rag1*^−/−^, nude and *Igh*^−/−^, suggest that T and B cells work together with the innate immune system to induce vasculitis, but neither cell type is indispensable for the induction of illness. The data also suggest that CCR2 modulates the role of T and B cells in the induction of vasculitis.

### Role of CCR2 in Treg depletion and Th17 expansion

**Figure 4:**
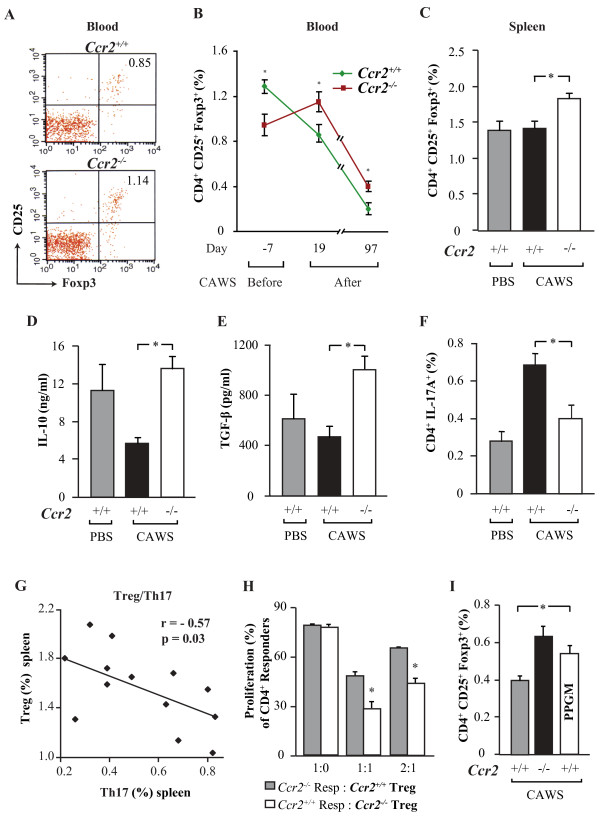
**Increased Treg and reduced Th17 cells in *Ccr2***^***−/− ***^**mice that received CAWS.** (**A**) Representative FACS of circulating Tregs gated on CD4^+^ cells (CD4^+^, CD25^+^, Foxp3^+^) from *Ccr2*^*+/+*^ mice (upper panel) and *Ccr2*^*−/−*^ mice (lower panel), after two cycles of CAWS. (**B**) Percentage of Treg in circulation in *Ccr2*^*+/+*^ mice and *Ccr2*^*−/−*^ mice, 1 week prior (day −7), day 19 and day 97 post-CAWS administration. (**C**) Quantification of Tregs in freshly isolated splenocytes derived from *Ccr2*^*+/+*^ mice and *Ccr2*^*−/−*^ mice that received a full cycle of CAWS. (**D**-**E**) Levels of IL-10 and TGF-β in cell culture supernatants of splenocytes stimulated with anti-CD3/CD28 for 60 hours in *Ccr2*^*+/+*^ and *Ccr2*^*−/−*^ mice, 10 days after the first cycle of CAWS administration. (**F**) Percentage of Th17 cells (CD4^+^, IL-17A^+^) in spleen detected by FACS in *Ccr2*^*+/+*^ and *Ccr2*^*−/−*^ mice, after full cycle of CAWS. (**G**) Negative correlation between circulating Tregs and Th17 cells in splenocytes cultures. (**H**) Treg suppression assay showing the percentage of proliferation of CD4^+^ responder cells from *Ccr2*^*−/−*^ mice cultured with Treg from *Ccr2*^*+/+*^ mice (grey bar) and CD4^+^ responder cells from *Ccr2*^*+/+*^ mice cultured with Treg from *Ccr2*^*−/−*^ mice (open bar) at different ratios (CD4^+^ responder : Treg; 1:0, 1:1 and 2:1); analyzed, after a short cycle of CAWS injections. (**I**) FACS analysis in blood showing the percentage of Treg in *Ccr2*^*+/+*^, *Ccr2*^*−/−*^ and PPGM treated *Ccr2*^*+/+*^ mice for 19 days, after one cycle of CAWS. Each bar represents the mean ± SE from a representative experiment with 6–8 mice per group.

To study the role of Treg (CD4^+^, CD25^+^, Foxp3^+^) in this model of aortic/coronary vasculitis after CAWS administration, we compared the circulating levels of Treg in *Ccr2*^*+/+*^ and *Ccr2*^*−/−*^ mice. We found that after two cycles of CAWS, the percentage of Treg analyzed by FACS were significantly increased in *Ccr2*^*−/−*^ compared to *Ccr2*^*+/+*^ mice (Figure 4A). Having found that Treg numbers were elevated after CAWS administration in *Ccr2*^*−/−*^, we decided to evaluate if these quantities remained constant or changed between WT and *Ccr2* null mice before and after disease induction. Examination of Tregs at different time points including 7 days prior to injection of CAWS, as well as before and after the second CAWS cycle revealed that CAWS injection in *Ccr2*^*+/+*^ mice resulted in a progressive reduction of Tregs in circulation; however, we observed a significant increase of these cells in *Ccr2*^*−/−*^ mice after disease induction, and that these numbers remained elevated during the course of the disease in *Ccr2*^*−/−*^ compared to WT mice (Figure 4B). Interestingly, prior to CAWS injection, *Ccr2*^*−/−*^ mice had a significantly lower proportion of Treg than *Ccr2*^*+/+*^ mice in circulation (Figure 4B).

Similarly, there was a higher proportion of Treg in the spleen of *Ccr2*^*−/−*^ mice compare to *Ccr2*^*+/+*^ mice 30 days after completing two cycles of CAWS (Figure 4C). Substantiating this observation further, we found that compared with CAWS-injected *Ccr2*^*+/+*^ mice, splenocytes from *Ccr2*^*−/−*^ mice stimulated with anti-CD3/CD28, released higher levels of IL-10 (Figure 4D) and active TGF-β (Figure 4E), cytokines that have been associated with Treg. Finally, there was an induction in the proportion of Treg in circulation after disease initiation, as well as the cytokines involved in Treg proliferation/differentiation, seen in *Ccr2* null mice. Based on this observation we decided to investigate if the presence of Treg in the locally affected areas (heart/coronaries) provided the protection seen in these animals compared to the WT. Treg cells were not detected in the heart using flow cytometry (CD4^+^CD25^+^Foxp3^+^) and RT-PCR (FoxP3) (data not shown). These results indicate that most likely the suppression conferred by Treg occurs distal to the inflamed regions (e.g. spleen).

Conversely, CAWS-injected *Ccr2*^*+/+*^ mice had a higher proportion of CD4^+^ and IL-17A^+^ cells (Th17) in the spleen, compared with *Ccr2*^*−/−*^ mice (Figure 4F). Supporting the notion that an imbalance between Treg and Th17 consequently leads to coronary vasculitis, we found a significant negative correlation between the proportion of Treg and Th17 cells in the spleen (Figure 4G). Nevertheless, we also found a reduced Th1 and Th2 response in the spleens of CAWS- injected *Ccr2*^−/−^ mice (Additional file 5), suggesting that increased Treg in the spleens of *Ccr2*^*−/−*^ mice may be associated with broader modulation of T cell responses.

Additionally, to determine the suppressor activity of Treg in the context of CCR2, functional assays were used in *Ccr2*^*+/+*^ and *Ccr2*^*−/−*^ mice. Treg from PBS-injected groups developed a clear suppressor activity characterized by decreased proliferation of responder CD4^+^ cells with different ratios. Interestingly, a stronger suppressor activity was found in *Ccr2* intact mice under different ratios of responder CD4^+^ cells compared with the *Ccr2* null mice (data not shown). Using the first cycle of CAWS for development of coronary vasculitis (19 days after CAWS administration), the same outcome was seen using a 1:1 proportion (responder:Treg), no differences were found at 1:2, and the opposite was found at 1:5 (data not shown). Finally, to compare the functional effect of *Ccr2* on the ability of Treg to suppress proliferation, Treg from *Ccr2*^*+/+*^ or *Ccr2*^*−/−*^ mice were cultured with responder CD4^+^ T cells of the opposite genotype. Notably, Treg from *Ccr2* null mice showed a significant suppressor activity against *Ccr2*^*+/+*^ responder T cells compared with regulatory T cells from *Ccr2*^*+/+*^ with *Ccr2*^*−/−*^ responder cells at different ratios (Figure 4H), indicating that absence of *Ccr2* can further enhance the suppressive capabilities of Treg.

Lastly, we evaluated a pharmacological approach to block CCR2 and its impact on the proportion of Treg. For this, propagermanium (PPGM) was used as a CCR2 blocker as has been demonstrated by Yokochi et al. [16] and others [17-20]. Remarkably, oral administration of PPGM significantly increased the percentage of Treg in circulation in *Ccr2* intact mice, compared to animals that did not receive treatment, following a trend similar to the one observed in *Ccr2* null mice (Figure 4I) and confirming our previous findings.

### Decreased immune response in CAWS-injected *Ccr2*^*−/−*^ mice

**Figure 5:**
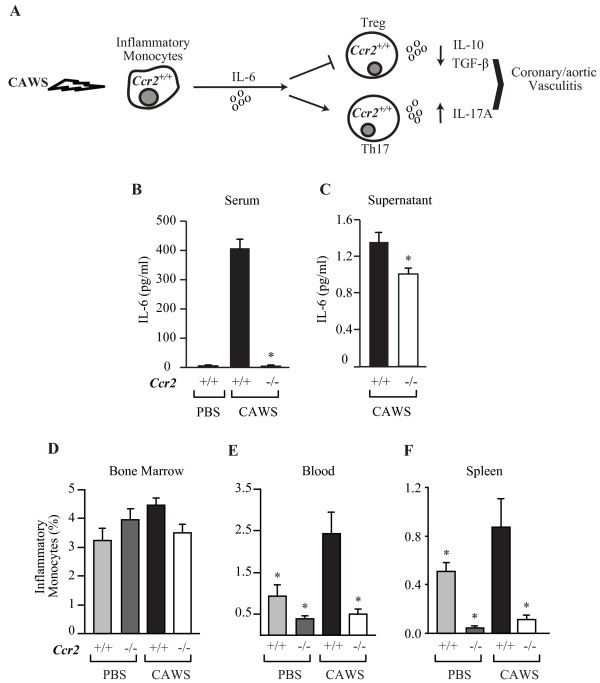
**Association of iMo mobilization from bone marrow to periphery with IL-6 increases after CAWS administration.** (**A**) Representative pathway showing the possible role of iMo releasing IL-6 after CAWS administration activating Th17 cells and inhibiting Treg cells in *Ccr2*^*+/+*^ mice. (**B**) Serum levels of IL-6 detected in *Ccr2*^*+/+*^ mice and *Ccr2*^*−/−*^ mice, after full cycle of CAWS. (**C**) IL-6 levels in cell culture supernatants of splenocytes stimulated with anti-CD3/CD28 for 60 hours in *Ccr2*^*+/+*^ and *Ccr2*^*−/−*^ mice, euthanized 10 days after the first cycle of CAWS administration. (**D**) FACS showing iMo (CD11b^+^, Ly6C^hi^, Ly6G^low^) in bone marrow, (**E**) blood and (**F**) spleen in *Ccr2*^*+/+*^ and *Ccr2*^*−/−*^ mice, 10 days after the first cycle of CAWS or PBS control.

*In silico* “pathways analysis” correlating CCR2, Th17 (IL-17A) and Tregs pointed at IL-6 as a molecular candidate linking the effect(s) of CCL2-CCR2-dependent signals on the Treg/Th17 balance (Figure 5A). Therefore, we measured serum levels of IL-6 in PBS- or CAWS-injected mice on days 10 and 30 after the first cycle of CAWS. The data revealed a systemic rise in the levels of IL-6 in *Ccr2*^*+/+*^ after CAWS injection that was ameliorated in *Ccr2*^*−/−*^ mice (Figure 5B). In agreement with the serum data, culture supernatants of splenocytes activated with anti-CD3/CD28 from CAWS-injected mice contained higher levels of IL-6 in *Ccr2*^*+/+*^ compared with *Ccr2*^*−/−*^ mice (Figure 5C). Moreover, highlighting possible links between reduced IL-6 production and lower proportion of Th17 cells in the spleen, we found a significant correlation between circulating levels of IL-6 and the percentage of Th17 cells in the spleen across all groups of mice (r=0.62, P=0.02, data not shown).

IMo also called M1 monocytes is a subtype of monocytes thought to be an important cellular source of IL-6 [21,22]. We found that CAWS injection resulted in mobilization of iMo into the periphery, as indicated by over a two-fold increase in the proportion of iMo in the blood and spleen of *Ccr2*^*+/+*^ CAWS-injected compared to PBS injected mice (Figure 5E and F). The proportion of iMo in the bone marrow (BM) of PBS injected *Ccr2*^*−/−*^and *Ccr2*^*+/+*^ mice was similar (Figure 5D). However, CAWS injected *Ccr2*^*−/−*^ mice had a lower proportion of iMo in the blood and spleen (Figure 5E-F) than CAWS-injected *Ccr2*^*+/+*^. Together these data suggest that although *Ccr2*^*−/−*^ mice have a similar proportion of iMo in the BM, these cells are not mobilized into periphery following the challenge with CAWS.

## Discussion

As seen in patients with KD, our murine model of coronary vasculitis was characterized mechanistically by the involvement of T and B cells [23] as well as the mobilization of iMo [24,25] with an increase of IL-6 levels [9,13]. Moreover, Treg/Th17 cell imbalance was correlated with a reduction of IL-10 and TGF-β together with an increase of IL-17 after CAWS administration as in KD [13,14]. Interestingly, genetic inactivation of CCR2, but not CCR5, is protective against CAWS induced-aortic and coronary vasculitis. Several lines of evidence support our findings that CCR2 plays a critical role in the pathogenesis of coronary vasculitis as possibly seen in KD. First, CCL2 levels, one of the main ligands for CCR2, are elevated in the serum [26] and urine [27] of patients with KD in the acute phase of illness; and this elevation is modulated by treatment [11]. Also, genetic evidence points towards a role for CCR2 in the pathogenesis of KD, as suggested by the association between KD and common genetic variants in the chemokine receptor gene-cluster CCR3-CCR2-CCR5 [5].

The role of lymphocytes and monocytes/macrophages has been described as a key factor in the pathogenesis of KD [9]. Also, in this study we show that T and B cells played a contributory role in the development of CAWS-induced vasculitis, as suggested by the decreased incidence of illness in *Rag1*^*−/−*^ mice. However, innate immune responses play a critical role as 50% of the *Rag1*^*−/−*^ mice still developed a less severe form of the illness. Indeed, selective absence of B or T cells was not associated with significant protection, indicating that in this experimental model the interaction between these two cell types and the innate immune response provides a high degree of redundancy. In our study, the development of vasculitis was likely related to an imbalance between inflammation and immune regulation, triggered by innate immune factors such as IL-6. This cytokine has a pivotal function for dictating whether T cells differentiate into Treg or Th17 cells [13,28,29]. In the presence of TGF-β and IL-6, precursors differentiate into Th17 cells, but when only TGF-β is present will they differentiate into Treg [30]. Thus, IL-6 was likely to inhibit the generation of Treg and induced the production of IL-17, a potent pro-inflammatory cytokine. Additionally, levels of mediators commonly released by Treg, such as IL-10 and TGF-β, were significantly elevated in *Ccr2*^*−/−*^ mice.

In CAWS-injected *Ccr2*^*+/+*^ mice, we found a significant depletion of Treg in the periphery that coincided with an increased proportion of Th17 cells in the spleen and elevated circulating levels of IL-6. Notably, *Ccr2*^*−/−*^ mice had lower circulating levels of IL-6 compared to *Ccr2*^*+/+*^ mice and interestingly; *Ccr2*^*−/−*^ mice had a higher proportion of circulating Treg after CAWS. In addition, the important role of *Ccr2* to control Treg function and proliferation in this model was uncovered by the fact that: i) *Ccr2*^*−/−*^ Treg had a higher suppressor activity on WT responder T cells and ii) *in-vivo* blocking of CCR2 increased the proportion of Treg in circulation.

Collectively, these data suggested a mechanistic scenario by which this chemokine receptor was involved in the innate response to CAWS leading to the rise in IL-6 production that favored a Th17 cell response at the expense of Treg.

Three lines of evidence emphasize the importance of IL-6 in KD and give credence to the notion that this mediator may be a determinant of the Treg/Th17 imbalance in the pathogenesis of coronary vasculitis [29]. First, higher levels of IL-6 have been consistently reported in patients with KD during the acute phase of illness and serum levels of IL-6 return to normal control levels following successful treatment and parallels the duration of the fever [9,31].

Second, comparable to our findings in WT mice injected with CAWS, which showed a sustained loss of Treg, the proportion of Treg is lower during acute KD and tends to normalize after the administration of IVIG [32]. In addition, has been shown that IVIG induces not only the expression of CD4^+^CD25^+^FoxP3^+^ cells, but also the secretion of immunosuppressive TGF-β and IL-10 [33]. Interestingly, the protective phenotype related with *Ccr2*^*−/−*^ mice, was associated with an increase in regulatory T cells, TGF-β and IL-10, and a reduction of IL-6 after CAWS administration.

Finally, supporting the role for Th17 responses in KD, serum IL-17 levels has been shown markedly elevated in patients with acute KD and positively correlated with IL-6 levels [34]. Importantly, IL-17 levels gradually decreased in the subacute phase [13,34].

What was the cellular source of IL-6 in mice injected with CAWS? In line with our findings in the CAWS-induced vasculitis, a growing consensus exists that one of the main pathogenic factors in KD is the activation of monocytes/macrophages [9,25]. For instance, during the acute phase, patients with KD have a significant increase in the absolute numbers of CD14^+^ monocytes [35], as well as in the percentage of CD14^+^CD16^-^ monocytes, the human correlate of mouse iMo. This increase is quite specific to KD and severe bacterial infections, but not to other febrile illness such as pneumonia, infectious mononucleosis, or anaphylactoid purpura [35]. CD14^+^CD16^-^ cells also trigger efficient immune responses [36]. Both, in humans and mice, iMo release high levels of pro-inflammatory cytokines, including IL-6 [37]. iMo are directly influenced by CCR2 i.e., cell activation, and indirectly, i.e., regulation of cell migration [36]. We found that CAWS injection promoted a CCR2-dependent emigration of iMo from the BM to periphery (i.e., blood, spleen). Increased availability of iMo in the periphery creates a readily available cellular source of IL-6. These findings were not unexpected considering the elegant work from Serbina *et al*., and others [36], indicating that CCR2 is required for the emigration of iMo from the BM into the periphery.

Some limitations need to be considered. First, no animal model can recapitulate all the features of KD, including age of onset [38]. Second, the assessment of T cell responses in peripheral blood of patients with KD or in the spleen of CAWS-injected mice prove informative; and the ongoing T cells dynamics that may be present in KD at the vascular wall, may not fully parallel our model, where we see a systemic ongoing immune activation. Additional experiments are needed to directly demonstrate the role for IL-6, Treg and Th17 in CAWS-induced vasculitis via antibody neutralization, genetic inactivation or cell expansion/depletion.

## Conclusion

Collectively, our findings provide novel insights into the role of CCR2 in the pathogenesis of vasculitis as seen in KD and other types of vasculitis, and highlighting novel therapeutic targets specifically for individuals resistant to first-line treatments.

## Methods

### Mice, *Candida albicans* water soluble (CAWS) and induction of vasculitis

C57BL/6J wild type (WT) and knockout mice (*Ccr2*^*−/−*^, *Ccr5*^*−/−*^, Nude, *Rag1*^*−/−*^ and *Igh*^*−/−*^) received CAWS [39] by injection (described in Additional file 1). In some experiments, animals only received the first cycle of CAWS. All mice were purchased from Jackson Laboratories (Bar Harbor, ME) and kept under pathogen-free conditions. The Institutional Animal Care and Use Committee of the UTHSCSA approved all protocols. CAWS was obtained as previously described [39].

### Histological evaluation

For this analysis we followed protocols previously described [40]. Fixed hearts were embedded in OCT and sectioned. 5–8 μm thick serial sections were collected every 20 μm, stained with hematoxylin and eosin (H&E) and examined by light microscopy. Then, for quantitative evaluation of vascular inflammation, we divided the area of the aortic root and coronary arteries into five segments that included: right coronary artery (RCA), left coronary artery (LCA), right coronary cuspid (RCC), left coronary cuspid (LCC), and non-coronary cuspid (NCC). Incidence was defined as having one or more inflamed areas. Also, we measured the area of inflammation surrounding the aortic root and coronaries as a proxy for disease severity using the ImageJ software (NIH, Bethesda).

### ELISA and immunostaining

For coronary and aortic analysis, macrophages were immunostained with the ER-HR3 antibody as previously described [40]. Area of infiltrating monocytes was quantified using ImageJ software (NIH).

Immunolabeling for MPO on tissues was conducted using a mouse MPO ELISA kit (Hycult Biotechology B.V, Netherlands). IgG1 and IgG2a antibodies against CAWS were measured in serum following a previously described protocol [41], but used CAWS as the antigen (10 μg/ml). Both, MPO and antibodies against MPO were analyzed in serum following the manufacturer’s protocols. ELISA for IL-10 (eBiosciences, San Diego) and TGF-β (R&D Systems, Minneapolis) were performed according to the manufacturer’s directions.

### FACS (fluorescence-activated cell sorting)

Cells from blood, bone marrow (BM), spleen and heart were used for staining. Leukocytes in the heart were harvested by digestion of tissue compromised of the root of the aorta and portions of the auricular and ventricular tissue, as previously described [42]. Tregs in whole blood, spleen and heart were stained with CD4, CD25 and Foxp3 antibodies following manufacturer’s instructions (eBiosciences). Antibodies for CD4, CD11b, Ly6C, Ly6G and I-A^b^ were purchased from BD Biosciences (San Jose). Events were acquired in a FACScalibur and data was analyzed in CellQuest-PRO (BD Biosciences). Antibody combinations used are presented in (Additional file 6: Table S1).

### RNA extraction and real-time PCR (RT-PCR)

Total RNA was extracted from the upper third portion of the heart, which included the root of the aorta and the coronaries, using the TRIzol reagent (Life Technologies) following manufacturer’s protocol. High capacity cDNA reverse transcription kit with RNase inhibitor (Applied Biosystems, Austin) was used on 500 ng of total RNA. A total of 125 ng cDNA was used for RT-PCR using Taqman primer and probe sets for FoxP3-FAM (Mm00475162_m1) and β-actin-VIC [(Mm00607939_s1) primer limited] (Applied Biosystems). cDNA samples were run in triplicate along with normal positive (spleen), negative (pancreas) and non-template controls. Real-time quantitative PCR was done with the SsoFast probes supermix (Bio-Rad, Hercules) in a CFX96 RT-PCR system (Bio-Rad). Threshold cycles were determined using the CFX Manager software v1.6. Changes in expression were calculated using the 2^-ΔΔCt^ method normalized to β-actin expression.

### Blockage of CCR2 by PPGM

In some experiments, *Ccr2* was blocked by oral administration of PPGM (Sigma-Aldrich, St. Louis) at a dose of 8mg/kg/day in the drinking water, for 30 days starting from the day when the first cycle of CAWS was injected.

### In-vitro suppression assay

CD4^+^CD25^+^ Treg and CD4^+^CD25^-^ responder T cells were isolated from pooled spleens of CAWS-injected WT and *Ccr2*^−/−^ mice, using the CD4^+^CD25^+^ regulatory T cell isolation kit with the AutoMACS (Miltenyi Biotec, Auburn) following manufacturer’s directions. Responder T cells were labeled with the CFSE cell proliferation kit (Life Technologies, Carlsbad) according to the kit protocol. Depleted CD4 cells obtained from the positive fraction during the first step of the regulatory T cell isolation, were used as feeder cells after treatment with 50ug/ml mitomycin (Sigma-Aldrich) during 45min, followed by three washes with RPMI. CD4^+^CD25^-^ responder cells (5×10^4^ cells/well initial number) were stimulated with 1ug/ml of soluble anti-CD3 (BD Biosciences) and syngenic feeder cells (5×10^4^ cells/well). CD4^+^CD25^+^ Treg (5×10^4^ cells/well) were added to the corresponding wells to the above cultures, and cells were incubated at 37^o^ for 72hrs. Each ratio of responder:Treg cells was run in triplicate. After 72hrs, cells were collected, washed and analyzed by FACS as described above. Proliferation gates were determined from wells where responder T cells lacked Treg, and from wells where responder T cells were cultured alone without stimulation (CD3 and feeder cells).

### Immune cell transfers

Isolation of untouched T and B cells from spleens derived from *Ccr2*^+/+^ or *Ccr2*^−/−^ mice were done using the Pan T cell isolation kit and the B cell isolation kit from Miltenyi Biotec. Cell purifications were performed with the AutoMACS (Miltenyi Biotec) according to the manufacturer’s directions. Levels of purity post-purification were determined by FACS and found to be above 90% for each cell population. Recipient mice received 1×10^6^ B and/or T cells via tail vein injection. To verify the reconstitution of T and B cells in each mouse, we stained the cells from the blood and spleen with CD4 and CD19 antibodies at the time of the sacrifice for FACS analysis. Recipient mice had higher percentages of T and/or B cells compared to PBS-treated mice; however no differences in the degree of reconstitution occurred between the recipients of *Ccr2*^*+/+*^ or *Ccr2*^*−/−*^ cells.

### Statistical analysis and data modeling

Data represent the mean ± SD. Groups were analyzed with Stata (StataCorp) or SPSS statistical software. According to the number of groups and the distribution (normally distributed or not), non-paired t test, one-way ANOVA, Kruskal-Wallis, Mann–Whitney, or Fisher’s exact tests were performed. Statistical significance was accepted at p< 0.05.

## Abbreviations

BM: Bone marrow; CAWS: *Candida albicans* water-soluble cell wall extracts; CCL2: CC chemokine ligand 2; CCR2: CC chemokine receptor 2; H&E: Hematoxylin and eosin; iMO: Inflammatory monocytes; IVIG: Intravenous immunoglobulin infusion; KD: Kawasaki disease; LCA: Left coronary artery; LCC: Left coronary cuspid; MPO: Myeloperoxidase; NCC: Non-coronary cuspid; PPGM: Propagermanium; Treg: Regulatory T cells; RCA: Right coronary artery; RCC: Right coronary cuspid; WT: Wild type.

## Competing interests

The authors declare that they have no competing interests.

## Authors’ contributions

All authors have read and approved the final manuscript. **HGM**: Study coordination, carried out immunoassays, bone marrow transplant, analysis and interpretation of data and drafted the manuscript. **MPQ**: Participate in design of the study, analysis and interpretation of data, critical review and drafted the manuscript. **FJ**: Immunoassays, collection of data, statistical analysis, bone marrow transplant and revision of the manuscript. **CE**: Collection of data, immunoassays, statistical analysis. **KMC**: Collection of data, immunoassays, animal maintenance and revision of the manuscript. **KS**: Participate in the design of the study and the creation of special reagents for the study. **NM**: Participate in the design of the study and the creation of special reagents for the study. **NO**: Participate in the design of the study and the creation of special reagents for the study. **SKA**: Participate in the design of the study and provided critical review of the manuscript. **SSA**: Acquisition of funding, conceived the study and supervision of research group, participate in design, critical review and final approval of manuscript. All authors read and approved the final manuscript.

## Supplementary Material

Additional file 1 **Figure S1.** Protocol for coronary/aortic inflammation in mice using CAWS. A full cycle of CAWS included two rounds of intra-peritoneal (I.P.) injections (1 mg/mouse/day for five consecutive days) administered four weeks apart as previously described^47-51^. Early experiments were conducted with a dose of 4 mg instead of 1 mg of CAWS per day. Results derived from either dose had identical disease incidence and severity. In some experiments, mice were sacrificed 10 days after the first five days of CAWS injections (cycle one). At this time point we were unable to identify ongoing inflammation in the coronary or aortic walls (data not shown). We identified inflammation clearly and consistently in 100% of mice 30 days after the completion of the first cycle. Click here for file

Additional file 2 **Figure S2.** Coronary and aortic analysis of macrophages. Coronary and aortic macrophages were immunostained with the ER-HR3 antibody in *Ccr2*^*+/+*^ and *Ccr2*^*−/−*^ mice including isotype control. Click here for file

Additional file 3 **Figure S3.** Serum levels of Myeloperoxidase (MPO). MPO levels were detected by ELISA after full cycle of CAWS in *Ccr2+/+* and *Ccr2−/−* mice. Click here for file

Additional file 4 **Figure S4.** Antibody against MPO (anti-MPO), IgG1 and IgG2a levels *Ccr2+/+* and *Ccr2−/−* mice after full cycles of CAWS. A. Serum antibodies against MPO detected by ELISA in *Ccr2+/+* and *Ccr2−/−* mice after two cycles of CAWS, including PBS control (values expressed as optical density). B-C. Serum levels of anti-IgG1 and anti-IgG2a, in *Ccr2+/+* and *Ccr2−/−* mice after two cycles of CAWS. Click here for file

Additional file 5 **Figure S5.** Th1 and Th2 response in spleen of *Ccr2*^*+/+*^ and *Ccr2*^*−/−*^ mice. Percentage of IFNγ (Th1) and IL-4 (Th2) in splenocytes after full cycle of CAWS in *Ccr2*^+/+^ and *Ccr2*^*−/−*^ mice. Each bar represents the mean ± SE from a representative experiment with 6–8 mice per group. Click here for file

Additional file 6 **Table S1.** Flow cytometric markers used to identify specific cell type. Click here for file
