# Supplementary figures and images for: Important role of CCR2 in a murine model of coronary vasculitis
Source: BMC Immunol. 2012 Oct 17;13:56. doi: 10.1186/1471-2172-13-56 (PMC3519555; doi:10.1186/1471-2172-13-56)

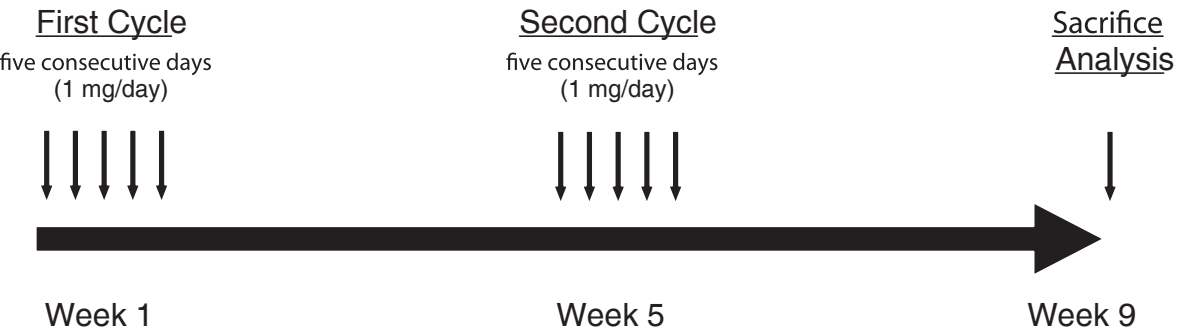

Additional Figure 1

Supplement: Additional file 1 — Figure S1. Protocol for coronary/aortic inflammation in mice using CAWS. A full cycle of CAWS included two rounds of intra-peritoneal (I.P.) injections (1 mg/mouse/day for five consecutive days) administered four weeks apart as previously described47-51. Early experiments were conducted with a dose of 4 mg instead of 1 mg of CAWS per day. Results derived from either dose had identical disease incidence and severity. In some experiments, mice were sacrificed 10 days after the first five days of CAWS injections (cycle one). At this time point we were unable to identify ongoing inflammation in the coronary or aortic walls (data not shown). We identified inflammation clearly and consistently in 100% of mice 30 days after the completion of the first cycle. [file 1471-2172-13-56-S1.pdf]

*Ccr2*<sup>+/+</sup>

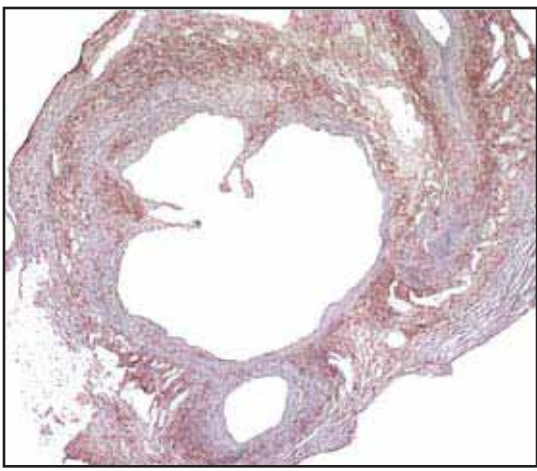

**ER-HR3**

*Ccr2*<sup>+/+</sup>

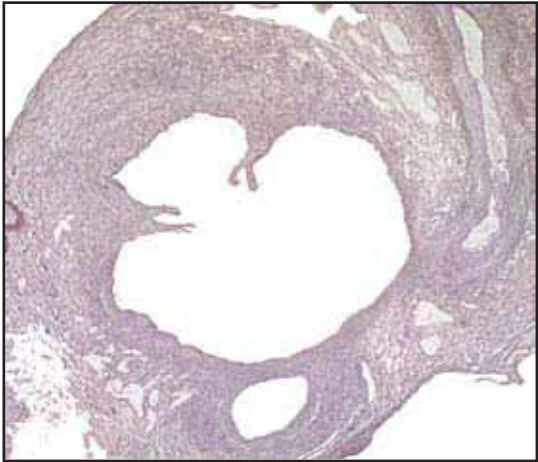

**ER-HR3 (isotype)**

*Ccr2*<sup>-/-</sup>

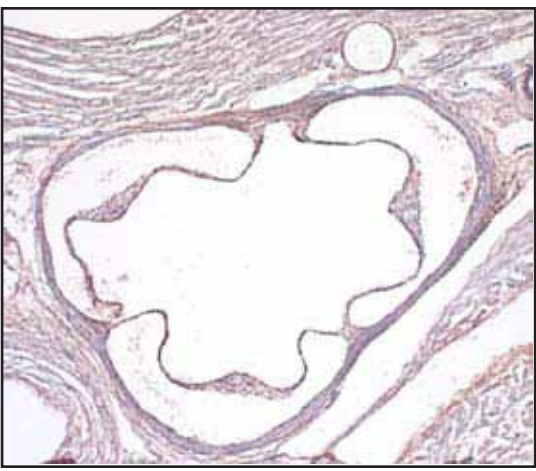

**ER-HR3**

Supplement: Additional file 2 — Figure S2. Coronary and aortic analysis of macrophages. Coronary and aortic macrophages were immunostained with the ER-HR3 antibody in Ccr2+/+ and Ccr2−/− mice including isotype control. [file 1471-2172-13-56-S2.pdf]

# Serum

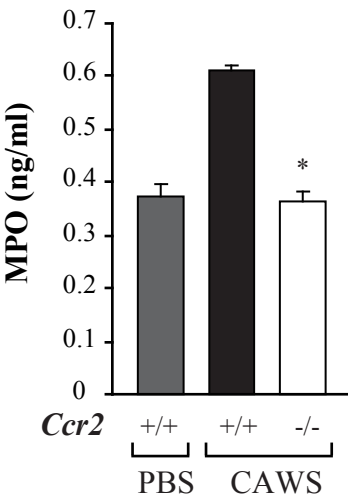

Additional Figure 3

Supplement: Additional file 3 — Figure S3. Serum levels of Myeloperoxidase (MPO). MPO levels were detected by ELISA after full cycle of CAWS in Ccr2+/+ and Ccr2−/− mice. [file 1471-2172-13-56-S3.pdf]

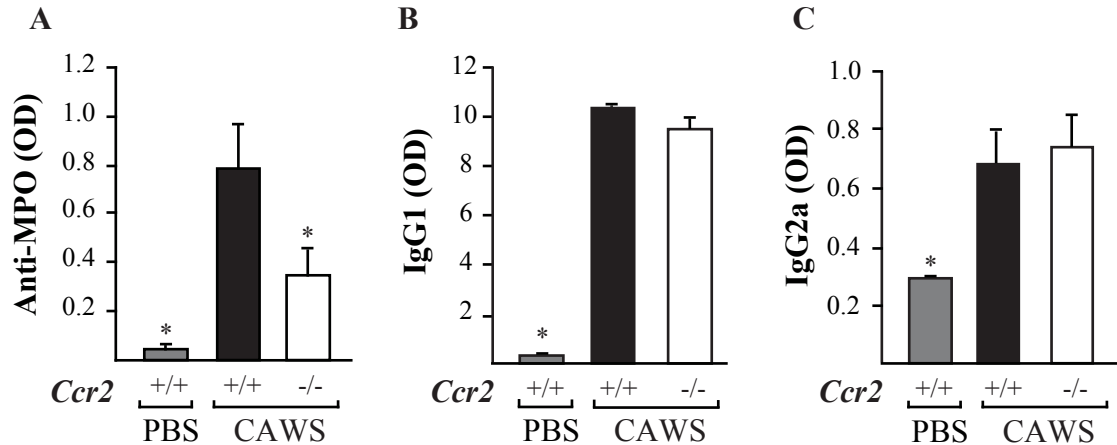

Additional Figure 4

Supplement: Additional file 4 — Figure S4. Antibody against MPO (anti-MPO), IgG1 and IgG2a levels Ccr2+/+ and Ccr2−/− mice after full cycles of CAWS. A. Serum antibodies against MPO detected by ELISA in Ccr2+/+ and Ccr2−/− mice after two cycles of CAWS, including PBS control (values expressed as optical density). B-C. Serum levels of anti-IgG1 and anti-IgG2a, in Ccr2+/+ and Ccr2−/− mice after two cycles of CAWS. [file 1471-2172-13-56-S4.pdf]

**A**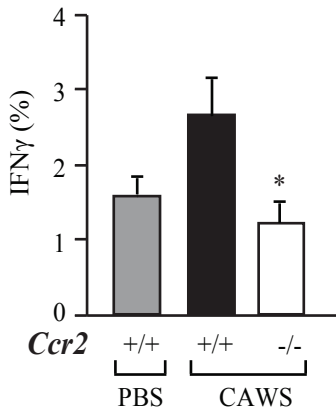**B**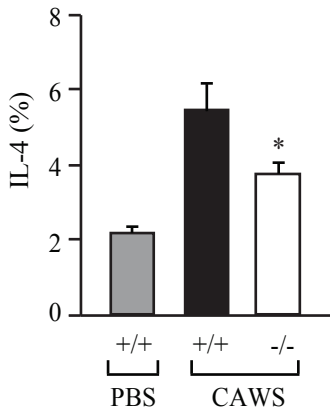

Additional Figure 5

Supplement: Additional file 5 — Figure S5. Th1 and Th2 response in spleen of Ccr2+/+ and Ccr2−/− mice. Percentage of IFNγ (Th1) and IL-4 (Th2) in splenocytes after full cycle of CAWS in Ccr2+/+ and Ccr2−/− mice. Each bar represents the mean ± SE from a representative experiment with 6–8 mice per group. [file 1471-2172-13-56-S5.pdf]
