# Supplementary material for: Important role of CCR2 in a murine model of coronary vasculitis
Source: BMC Immunol. 2012 Oct 17;13:56. doi: 10.1186/1471-2172-13-56 (PMC3519555; doi:10.1186/1471-2172-13-56)
Supplement: Additional file 6 — Table S1. Flow cytometric markers used to identify specific cell type. [file 1471-2172-13-56-S6.pdf]

**Additional Table**

Flow cytometric markers used to identify specific cell type

| Cell Type | Markers |
|---|---|
| Inflammatory monocytes | $CD11b^{+}$ $Ly6C^{high}$ $Ly6G^{interm.}$ |
| Neutrophils | $CD11b^{+}$ $Gr\text{-}1^{+}$ |
| Dendritic cells | $CD11c^{+}$ $CD11b^{+}$ $I\text{-}A^{b+}$ |
| Regulatory T cells | $CD4^{+}$ $CD25^{+}$ $Foxp3^{+}$ |
